# A large anastomosing hemangioma in the left lobe of liver

**DOI:** 10.1097/MD.0000000000046179

**Published:** 2025-11-28

**Authors:** Ning Tian, Yankun Zhao, Jianxian Liu, Jie Gan

**Affiliations:** a Department of Radiology, Shandong Provincial Third Hospital, Jinan, China.

**Keywords:** anastomosing hemangioma, case report, liver

## Abstract

**Rationale:**

Anastomosing hemangioma (AH) is a rare benign hemangioma that predominantly occurs in the genitourinary system and retroperitoneum, and with a low incidence rate in the liver. Most cases have difficulty making a clear diagnosis before surgery and are prone to misdiagnosis. This case report aims to enhance the understanding of the occurrence of rare AHs in the liver and reduce unnecessary clinical interventions.

**Patient concerns:**

A 55-year-old male presented with a lesion in the left lobe of the liver during physical examination, which was surgically removed 1 week later. All the liver-related tumor indicators of the patient were negative.

**Diagnoses:**

The preoperative imaging examination diagnosed the lesion as a vascular tumor, and the AH was confirmed by pathological examination.

**Interventions:**

The patient underwent laparoscopic resection of the left lobe liver tumor and abdominal exploration and routine computed tomography follow-up.

**Outcomes:**

The patient recovered well after surgery, and the computed tomography scan 4 months after surgery showed no recurrence of the lesion.

**Lessons:**

For a single lesion in the liver with imaging manifestations of vasogenic tumors, when the imaging features and laboratory examinations lack specificity, the possibility of an AH should be considered.

## 1. Introduction

Anastomosing hemangioma (AH) is a clinically rare subtype of benign hemangioma that was first reported and named by Montgomery and Epstein in 2009.^[1]^ AH is a rare disease that predominantly occurs in the genitourinary system and retroperitoneum, and its occurrence in the liver is even rarer.^[2]^ Here, we reviewed the clinical and pathological features of a large AH in the left lobe of the liver.

## 2. Case presentation

A 55-year-old male patient underwent physical examination that detected a lesion in the left lobe of the liver. He had no abdominal pain or distension, and no accompanying jaundice of the skin or mucous membranes. The patient had no history of hepatitis or blood transfusion. He had a history of hypertension for >10 years, >1 year after coronary artery balloon dilation surgery, and >10 years since the repair surgery of the left knee cruciate ligament. He had no history of smoking or excessive alcohol consumption, diabetes, drug abuse, exposure to industrial poisons, dust, radioactive substances, or family history of hereditary diseases. Vital signs were as follows: temperature (T) = 36.3℃, pulse = 80 beats per minute, respiration rate = 20 breaths per minute, blood pressure = 158/102 mm Hg, alpha-fetoprotein = 3.41 ng/mL, carbohydrate antigen 199 (CA199) = 2.78 U/mL, carcinoembryonic antigen = 1.05 ng/mL, and C-reactive protein = 3.5 mg/L.

## 3. Outcomes

### 3.1. Imaging findings

**Figure 1.**
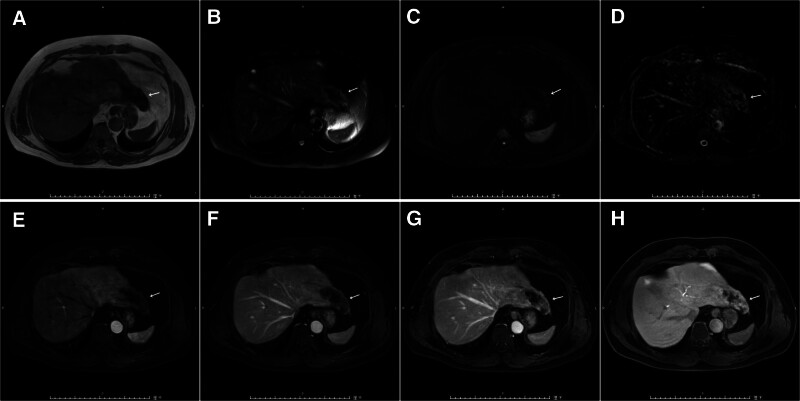
MRI manifestations of AH in the left lobe of the liver. (A) T1WI and (B) T2WI with fat suppress. (C) DWI (b = 800 s/mm²). (D) ADC map. (E) Arterial phase. (F) Portal venous phase. (G) Delayed phase. (H) Hepatobiliary phase. ADC = apparent diffusion coefficient, AH = anastomosing hemangioma, DWI = diffusion-weighted imaging, MRI = magnetic resonance imaging.

**Figure 2.**
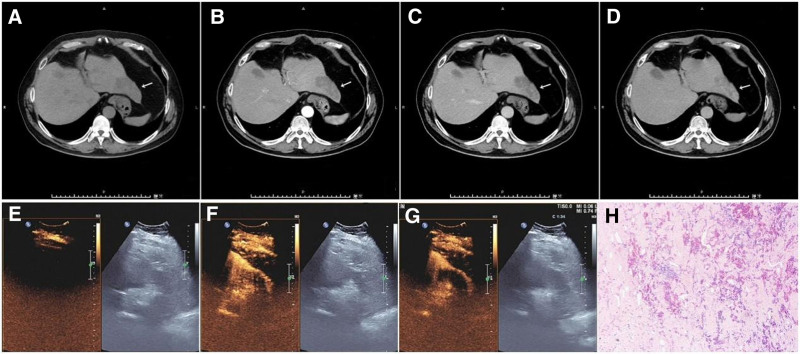
CT and CEUS manifestations of AH. (A) Plain CT scan. (B) Arterial phase. (C) Portal venous phase. (D) Delayed phase. (E) Arterial phase (CEUS). (F) Venous phase (CEUS). (G) Delayed phase (CEUS). (H) Pathological image. CEUS = contrast-enhanced ultrasound, CT = computed tomography.

Magnetic resonance imaging (MRI) showed an irregular abnormal signal lesion in the left lobe of the liver, with a clear boundary. The maximum cross-section was ~7.6 cm × 3.0 cm. T1WI and T2WI fat-suppressed sequences showed mixed signals. On diffusion-weighted imaging (b = 800 s/mm²), the edge showed slightly high signals and the signal on the apparent diffusion coefficient map was not low. Enhanced scanning showed marginal enhancement, and centripetal filling was observed in the hepatobiliary phase. Striped, non-enhanced areas were observed at the center (Fig. 1).The lesion appeared as a low-density mass on computed tomography (CT) (CT value = 42 HU). During enhanced scanning, there was mild marginal enhancement in the arterial phase, and centripetal filling gradually occurred in the portal venous and delayed phases; the highest CT value was ~71 HU (Fig. 2A–D). Contrast-enhanced ultrasound revealed focal enhancement in the arterial phase (Fig. 2E–G).The tumor was tentatively diagnosed as a vasogenic tumor.

### 3.2. Operative observations and follow-up

During the surgery, a tumor in the left lateral lobe of the liver was found upon exploration, and the tumor size was ~8 cm × 3 cm. The tumor was resected, and no abnormalities were found in the peritoneum or greater omental tissue. Subsequently, the left lateral lobe of the liver was resected. The intraoperative blood loss was ~20 mL. A reexamination was performed 4 months after the operation, and no signs of recurrence were found.

### 3.3. Pathological results

The pathological results showed an AH, and the blood vessels were in an anastomosing pattern accompanied by large-area hyaline degeneration. Vascular endothelial cells were hobnail-shaped. No obvious mitotic figures were observed and scattered extramedullary hematopoiesis was observed. Immunohistochemistry results were as follows: CD31 (vessel+), CD34 (vessel+), ERG (vessel+), SMA (+), S100 (−), SOX-10 (−), STAT-6 (−), MUC4 (−), CK (−), EMA (−), ER (−), PR (−), HMB45 (−), MelanA (−), CD61 (−), CD235a (+), MPO (−), and Ki67 (1%+).

Multiphase gadolinium-enhanced MRI showed a lesion (white arrow) in the left lateral lobe of the liver. On diffusion-weighted imaging (b = 800 s/mm²), restricted diffusion at the edge is observed (Fig. 1C). There is no obvious enhancement in the early arterial phase of enhanced scanning (Fig. 1E). Marginal enhancement is seen in the portal venous phase (Fig. 1F) and the delayed phase (Fig. 1G). Centripetal filling of the contrast agent is observed in the hepatobiliary phase (Fig. 1H), and strip-shaped non-enhanced areas are seen in the center.

Contrast-enhanced CT tomography shows a lesion (white arrow) in the left lateral lobe of the liver. After enhancement, punctate marginal enhancement was observed in different phases and centripetal filling of the contrast agent was observed (Fig. 2B–D). Contrast-enhanced ultrasonography showed focal enhancement of the lesion in the left lobe of the liver in the arterial phase, which gradually weakened and disappeared in the venous and delayed phases (Fig. 2E–G). Pathology (HE, ×100) showed that the blood vessels were in an anastomosing pattern, accompanied by large-area hyaline degeneration. The vascular endothelial cells were hobnail-shaped, and no obvious mitotic figures were observed (Fig. 2H).

## 4. Discussion

Anastomosing hemangioma (AH) is a rare subtype of hemangioma. This was first reported by Montgomery and Epsteinin 2009.^[1]^ The main affected population was middle-aged and elderly people, with a male-to-female ratio of ~ 1.8:1. AH predominantly occurs in the genitourinary system, such as the kidneys and testicles, and can also occur in the adrenal glands, gastrointestinal tract (including the liver), ovaries, paravertebral region, and other sites.^[3]^ Patients with AH usually have no related clinical symptoms and are generally detected incidentally during imaging examinations.

AH of the liver are rare. This was first reported in 2013,^[4]^ and its basic characteristics include anastomosing capillary-like blood vessels, nail-shaped endothelial cells, and a regular tumor boundary with expansive growth.^[4]^ Currently, most reports in domestic and foreign literature are case reports, and there are no reports on the incidence rate. In previous studies, single lesions in the liver were more common than multiple lesions,^[5]^ and lesions in the right lobe of the liver were more common in the left lobe.^[6–15]^ There were no specific clinical symptoms, and liver-related tumor indicators were negative.

Currently, there is no unified understanding of imaging diagnosis of hepatic AH in clinical practice. However, most hepatic AHs possess the imaging characteristics of general vasogenic tumors of the liver. In CT images, they mostly appear at low density. In MRI images, they are mostly isointense or hypointense on T1WI, and hyperintense on T2WI. Restricted diffusion was observed at the edges. On enhanced scanning, ring enhancement or marginal enhancement is observed in the arterial phase, and the enhanced area increases in the portal venous and delayed phases. No enhancement was observed at the center of the larger lesions. It is easily misdiagnosed as atypical hepatic cavernous hemangioma or primary hepatic angiosarcoma. The final clinical diagnosis depends on the pathology.

Morphologically, gross observations generally show that the tumor has a clear boundary, mostly without a capsule. The cut surfaces were gray or reddish-brown, meaty, and spongy. Microscopically, the basic lesion is a clearly demarcated sinusoid-like, cribriform, clustered, and anastomosing vascular network lined with hobnail-like endothelial cells, similar to the red pulp of the spleen. The cells had mild atypia but no mitotic figures, and the tumor usually did not invade the surrounding tissues. Simultaneously, it is accompanied by edematous or hyalinized sclerotic stroma, loose lobular structures, intravascular thrombosis, peripheral vascular dilation of the tumor, and venous hemangioma. Mature adipose tissue, fibrin thrombi, and extramedullary hematopoiesis can also be seen. Immunohistochemistry often shows positive expression of vascular endothelial markers (such as CD31, CD34, and ERG), and a low Ki67 index (<2%).^[16]^ In this case, the results showed that CD31, CD34, ERG, and SMA were positive and Ki67 was 1%+.

Currently, no unified treatment plan is available for hepatic AH. Most of the cases reported in the literature had an unclear preoperative diagnosis or were diagnosed with angiosarcoma and underwent surgical resection or puncture pathological examination. At present, there is no evidence indicating metastasis or recurrence after AH resection.^[16]^

As an individual case, whether the findings apply to a broader population is difficult to determine. We did not conduct long-term follow-up on the patient, which is also a drawback of our study.

In conclusion, AH of the liver is a clinically rare benign tumor of the liver, and its diagnosis depends on the pathology. For a single space-occupying lesion in the liver with imaging manifestations of vasogenic tumors, when the imaging features, clinical symptoms, and laboratory examinations lack specificity, the possibility of an AH should be considered, with the aim of preventing overdiagnosis and avoiding overtreatment.

## Author contributions

**Conceptualization:** Ning Tian, Jie Gan.

**Formal analysis:** Ning Tian, Jianxian Liu.

**Investigation:** Yankun Zhao, Jianxian Liu.

**Supervision:** Jie Gan.

**Visualization:** Ning Tian, Yankun Zhao, Jianxian Liu.

**Writing – original draft:** Ning Tian, Yankun Zhao.

**Writing – review & editing:** Jie Gan.
